# Screening and Identification of Indigenous Entomopathogenic Fungal Isolates from Agricultural Farmland Soils in Nile Delta, Egypt

**DOI:** 10.3390/jof8010054

**Published:** 2022-01-05

**Authors:** Alsayed Alfiky

**Affiliations:** 1 Genetics Department, Faculty of Agriculture, Tanta University, Tanta 31527, Egypt; alfiky@agr.tanta.edu.eg or alsayed.alfiky@unifr.ch; 2 Department of Biology, University of Fribourg, Rue Albert-Gockel 3, 1700 Fribourg, Switzerland

**Keywords:** biocontrol, Nile Delta, entomopathogenic fungi, *Beauveria bassiana*, *Metarhizium anisopliae*, *Spodoptera litura*, insect bait, *Galleria mellonella*, molecular identification

## Abstract

The compound negative impact of insect pests attacking agricultural ecosystems includes (i) direct yield losses from damaged crops, (ii) the economic cost of the attempt to prevent these losses and (iii) the negative short- and long-term hazard effects of chemical pesticides on human and environmental health. Entomopathogenic fungi (EMPF) are a group of microorganisms that represent the natural enemies of a number of crop pests, presenting an opportunity to harness their evolutionary fine-tuned relationship with their insect hosts as biocontrol agents in integrated pest management programs. The aim of this study was to establish an indigenous EMPF collection via the *Galleria mellonella* (greater wax moth) entrapment method from the soils of Nile Delta, Egypt. Obtained insect associated fungal isolates were bio-assayed for pathogenicity against the serious pest *Spodoptera litura* and *Tenebrio molitor,* and the seven outperforming isolates were selected for molecular identification and thermotolerance assay. Based on ITS sequence analysis and phylogeny, selected isolates were identified as *Beauveria bassiana* (four isolates), *Metarhizium anisopliae* (two isolates) and one isolate of *Cordyceps javanica*. The obtained results demonstrated (i) the efficacy of using insect baiting coupled with molecular identification and pathogenicity screening to isolate EMPF to control insect pests, and (ii) the availability of indigenous virulent EMPF in Nile Delta’s soil, which can be exploited for the development of sustainable crop protection strategies.

## 1. Introduction

The current pace of increase in world population, combined with limited—and, in many cases, declining—agricultural resources represent a major global challenge. Therefore, new multi-faceted sustainable agricultural strategies are required to meet the increasing demand for crop production [1]. Biological control in general and, in particular, biocontrol of insect pests is considered at the heart of such strategies in modern agriculture. Several factors influence the extent of crop losses from insect attacks such as environmental conditions, control strategy, plant resistance, alertness and the socioeconomic status of farmers [2]. Based on limited available data, it was suggested that between 18–20% of annual crop production at a value of more than US $470 billion is lost due to arthropod attacks [3]. Such catastrophic crop losses provided a golden opportunity for the insecticide industry to distribute and encourage using their chemical products promising to deliver the desired protection. Unfortunately, in a market driven by profitability before sustainability, chemical applications became the preferred and most widespread pest control strategy despite their negative impact on human and environmental health [4,5]. However, increasing awareness and interest in organic and sustainable agriculture in the last few decades, and the ability of entomopathogenic fungi (EMPF) to kill insects, have attracted the attention of environmentally friendly biocontrol strategies to counteract insect pests’ negative impact on crop yield and quality [6,7,8].

In the natural habitat, EMPF are a diverse group of insect-invading fungal pathogens that can effectively infect and regulate numerous insect pest populations [9,10,11]. In this regard, and to benefit from the evolutionary fine-tuned relationship between EMPF and their host insects, it is necessary to obtain and screen potential EMPF isolates and characterize their important properties such as insect host range, environmental persistence, plant growth impact and other important traits. Several approaches could be used to obtain potential EMPF such as (i) direct isolation from insect cadavers showing signs of fungal infestation, (ii) isolation from the soil (either directly on selective media or indirectly using insect baiting), and (iii) isolation from associated plants where some EMPF can establish an endophytic relationship with their host [6,12,13]. In this regard, obtainment of indigenous EMPF adapted to the local or specific environment (host insect ecotypes, temperature, humidity, flora, fauna, soil type and pH) for potential use as biocontrol agents (BCAs) have been proven to be more effective to control local pests of those areas compared to using non-native based applications [14]. Crop intensification and excessive use of chemical pesticides might pose limitations on environmental biological diversity in soil microbiota, including EMPF. The latter could be negatively affected by these practices, reducing their efficacy against their host insects. Therefore, it is of paramount importance to obtain updated knowledge of available EMPF in local agricultural ecosystems and their efficacy against important pests of these areas for the prospect of a development of bioinsecticides adapted to local conditions.

Egypt’s total land area is one million km^2^ with a total population of over 100 million inhabitants and the spatial distribution of both urban and agricultural areas reveal that they are concentrated in the Nile Valley and its Delta, which comprise only 4% of the total area of the country [15,16]. The Delta has a typical wave dominated, arc-shape and elevation ranging from 17 m above sea level in the south, which is decreasing gradually to 1 m in the northern frontier. The densely populated Nile Delta represents a cornerstone for Egypt’s food security and stability as it represents about 63% of its arable lands, 65% of agricultural production and almost 50% of the country’s population [16,17].

Soil is a natural reservoir for diverse microbial communities whose interactions collectively affect soil health and impact plant growth and physiology [18]. Similar to other soil microbiota, EMPF are influenced by both biotic and abiotic factors [19,20]. Therefore, it is of high importance to screen for indigenous and locally adapted EMPF strains with potentially promising activities against insect pests threatening crop production [21]. Several previous studies aimed to isolate pathogenic fungi, including EMPF, from Egyptian soils and their results reported the occurrence and distribution from various regions and soil systems [22,23,24,25,26]. This study aimed to identify naturally occurring EMPF from the soil of agricultural ecosystems from Nile Delta following the method of “insect baiting” using *Galleria mellonella* L. (Lepidoptera: Pyralidae) for its reported sensitivity and vulnerability against EMPF infections [27]. Thereafter, 2 rounds of pathogenicity screening were performed against *Spodoptera litura* (Fabricius) (Lepidoptera: Noctuidae) and *Tenebrio molitor* L. (Coleoptera: Tenebrionidae) to confirm virulence of obtained isolates. Molecular identification and phylogenetic analysis of seven isolates were performed to reveal their taxonomic position, and thermotolerance analysis was performed as a parameter for environmental persistence. It is believed that for the successful development of biopesticides for pest control, obtainment and assessment of potential biocontrol agents (BCA) are necessary first steps. Our experimental procedure in this work might provide a useful and standard tool in biological pest control.

## 2. Materials and Methods

### 2.1. Soil Sampling

Soil samples of approximately 100 g were collected at a depth of 5–10 cm from soil surface from four regions in three governorates (Gharbia, Kafr El-Shaikh and Damietta) in Nile Delta. Sampling sites were traditional farmlands and/or orchards located near the cities of Tanta (30°49′ N 30°58′ E), Kafr El-Zayat (30°47′21″ N 30°47′18″ E), Kafr El-Shaikh (31°08′ N 30°59′ E) and Damietta (31°23′ N 31°46′ E) in the Nile Delta region north of Egypt. Samples were obtained from farmlands that are regularly cultivated with annual crops such as corn, alfalfa, potato, onion, cotton, and/or from citrus and apple orchards (only from one region, Tanta). Samples were placed individually in clean plastic zip lock bags and stored at 4 °C until processed. In the laboratory, all the samples from each location were mixed together and homogenized to (i) maximize the geographical coverage of the sample and (ii) minimize the required experiments ((15 samples from nearby farmlands/orchards were pooled and homogenized into 1 sample) × 4 locations (different villages) per region × 4 regions = 16 final soil samples). Soil samples were collected in two-week interval in July in the mornings between 8:00–11:00 am with an average maximum temperature of 37 °C during daytime. Samples were processed simultaneously within one week of the last sample collection.

### 2.2. Entrapment of Entomopathogenic Fungi by Insect Baiting

A laboratory reared strain of *G. mellonella* maintained at 25 ± 2 °C with 70% relative humidity (RH), a 12:12 (L:D) photoperiod and fed on an artificial diet [28] was used for entrapment of soil-associated fungi using insect baiting technique as previously described [29,30]. Briefly, collected soil samples were allowed to dry at room temperature for 3 days on a laboratory stall (to avoid infections by entomopathogenic nematodes) and pooled together after removing large debris/stones and sieving through an aperture sieve to obtain homogenous texture. Soil samples (3 replicates per sample) were re-moistened using sterile distilled water and transferred into clean plastic trays (33 cm × 25 cm × 5 cm) leaving 2 cm of empty headspace between the soil surface and lid before introducing the insects. Thereafter, five random L6–L7 larvae of *G. mellonella* were added to each tray covered with lids containing small holes (1–2 mm in diameter) to allow for air exchange and ventilation of the larvae. The trays were incubated for 10 days at 25 °C in the dark in the laboratory and the larvae were examined daily after the fifth day of incubation. In the control treatment, the larvae were added to autoclaved soil sample. The trays were gently inverted and rotated to increase the probability of an encounter between the larvae and soil entomopathogenic fungi. Insect survival was monitored regularly; dead larvae were picked up carefully, washed with 1% Na-hypochlorite and transferred to sterile Petri plates (35 mm × 10 mm) lined with moistened filter paper. The plates were parafilmed and incubated in a dark chamber at room temperature to observe the development of mycosis and possible infestation by EMPF. Insect cadavers showing signs of mycosis were examined using a stereomicroscope and used to obtain fungal isolates on Sabouraud dextrose agar medium (BD Difco, Detroit, MI, USA) supplemented with 50 mg/mL chloramphenicol and 30 mg/mL streptomycin sulfate. Obtained isolates were purified using single spore technique [31] and 2–3-week-old fungal cultures were examined morphologically and microscopically before proceeding to molecular identification. Pure monosporic cultures were maintained as glycerol stock in −80 °C for future work.

### 2.3. Pathogenicity Screening

Obtained fungal isolates were cultured on PDA medium (90 mm × 15 mm) for 10–14 days until sporulation. Spore suspensions (1 × 10^8^ spore/mL) were prepared in sterile water with 0.02% Tween 80 and, using sterile glass beads, 250 µL of spore suspensions, were spread on PDA plates (150 mm × 25 mm) and incubated for two weeks. Thereafter, five random fourth instar larvae of *S. litura* were introduced to each fungal plate and incubated at 25 °C for 24 h. During this short window of group exposure to potential EMPF, *S. litura* larvae exhibited little or no cannibalism. Each larva was then individually transferred to new plates with a semi-artificial diet to avoid the risk of cannibalistic behavior [32]. As a control treatment, larvae were introduced to an uninoculated PDA plate and were incubated under the same conditions. Insects’ mortality was examined and recorded at 3, 6- and 10-days post infection (dpi) and insect cadavers were removed onto clean plates to monitor signs of mycosis to confirm fungal infestation as a probable cause of death. Mortality rates in EMPF treated larvae were calculated and corrected against their respective controls using Abbott’s formula [33] to account for natural mortality in control treatments.

### 2.4. Molecular Identification and Phylogenetic Analysis

Fungal genomic DNA was obtained by culturing monosporic isolates at 28 °C on sterile cellophane membrane overlaid on PDA plates. Fungal cultures were easily detached from the cellophane membrane using sterile spatulas and genomic DNA was extracted using DNeasy^®^ Plant Mini Kit (Qiagen, Hilden, Germany). The nuclear ribosomal internal transcribed spacer (ITS) region was amplified with forward primer ITS1 (5′-TCCGTAGGTGAACCTGCGG-3′) and reverse primer ITS4 (5′-TCCTCCGCTTATTGATATGC-3′) in 50 μL PCR reactions [34]. Reaction conditions were as follows: initial denaturation at 94 °C for 3 min, followed by 34 cycles of DNA denaturation at 94 °C for 45 s, primer annealing at 55 °C for 45 s, and DNA synthesis extension at 72 °C for 45 s, followed by a single step of final extension at 72 °C for 10 min. Obtained PCR products were visualized on 1.5% agarose gel with 1× tris TAE buffer and E-Gel Sizing DNA Ladder (Invitrogen) was used as a reference to estimate amplicon size. PCR products were prepared for sequencing by purification with a GenElute PCR purification kit (Sigma-Aldrich, St. Louis, MO, USA) following the manufacturer’s protocol. After the PCR products were sequenced using Sanger sequencing protocol, the obtained sequences were blasted against the NCBI database to identify the isolates. Published sequences representative for fungal ITS regions (Supplementary Table S1) were retrieved [13,35] and aligned with obtained sequences (Supplementary Table S2) using MEGA11 software (https://www.megasoftware.net, accessed on 1 May 2021). A phylogenetic tree was constructed with the maximum likelihood algorithm and Jukes–Cantor model and validated with 1000 bootstrap replicates.

### 2.5. Second Round of Bioassays

Based on the results of the first pathogenicity screening against *S. litura*, and the molecular identification results, the seven best performing isolates belonging to the genera *Beauveria, Metarhizium* and one isolate of *Cordyceps javanica* were subjected to a second pathogenicity bioassay against *S. litura* as well as a fourth instar larvae of *T. molitor* following the previously described procedure. The number of dead larvae was counted and recorded in 24 h intervals, and the dead cadavers were recognized as dead due to a fungal infestation upon visual observation of mycosis in the cadaver’s epidermis.

### 2.6. Thermotolerance and Conidial Germination Assay

To examine conidial stability and germination against elevated temperature, conidial suspensions (1 × 10^7^ conidia/mL) were prepared using hemocytometer from 2-week PDA plates cultured at 25 °C. The suspensions were then aliquoted onto 25 µL volumes in sterile PCR tubes, placed in a thermal cycler adjusted to 45 °C and removed in 45 min intervals (0, 45, 90, 135 and 180 min). Thereafter, 5 µL of heat-exposed conidia were plated onto the PDA medium directly without spreading and incubated for 16 h before evaluating germination rates. Control treatment consisted of conidial suspensions that were not heat-exposed. The germination rate for each isolate was calculated by comparing germination percentage to the relative control. On average, 100 conidia were examined for each time-point and the experiment was performed twice.

### 2.7. Statistical Analysis

Results were analyzed using GraphPad Prism, version 8 (GraphPad Software Inc., La Jolla, CA, USA). Results are presented as means ± standard deviation (SD) and one-way ANOVA was used with a Tukey HSD multiple comparison test. Differences between means (*p* < 0.05) were considered significant.

## 3. Results

### 3.1. Isolation and First Pathogenicity Screening of Insect Associated Fungal Isolates

**Figure 1:**
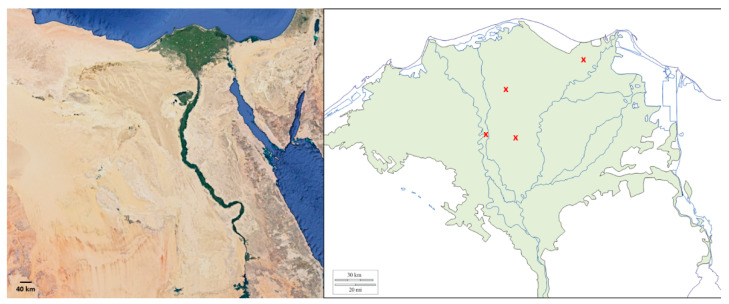
The map of Egypt to the left (Google Earth, accessed on 1 May 2021. 26°41′46″ N 30°47′53″ E) shows the green narrow strip of the Nile Valley and its Delta as the vital food basket for Egypt’s 100-million population. The (×) signs on the map of Nile Delta to the right (https://d-maps.com, accessed on 1 May 2021) indicate approximate soil sampling regions.

Presence of EMPF was confirmed in all sampling regions in this study (Figure 1). In the larvae baiting, 240 larvae (plus control treatments) of *G. mellonella* were used and after 10 days of baiting, 48 cadavers developed possible signs of fungal infestations and mycosis. In total, 39 insect associated fungal isolates were obtained using this technique. The spatial distribution of potential indigenous EMPF isolates revealed that out of the total 39 isolates, the highest number of isolates (13) was obtained from the soil of farmlands around the city of Damietta and Kafr El-Shaikh (13 isolates each), followed by 8 and 5 from the soils of farmlands around the cities of Tanta and Kafr El-Zayat, respectively.

**Figure 2:**
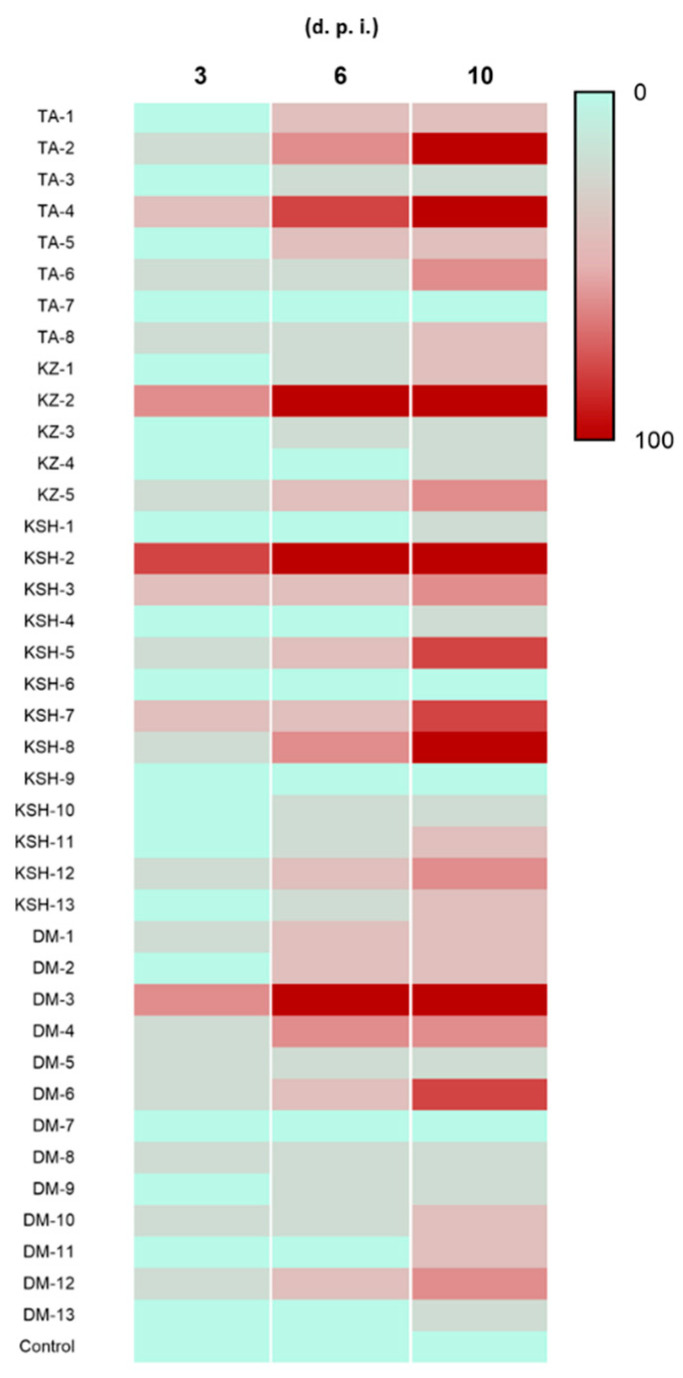
Insect killing heatmap of 39 insect associated fungal isolates against *S. litura* larvae at 3, 6- and 10-days post infection (d.p.i.). Isolates were obtained using the *G. mellonella* baiting technique and were isolated from different regions in Nile Delta north of Egypt.

The 39 obtained isolates were subjected to a first round of pathogenicity screening against *S. litura* to confirm that potential EMPF isolates were the cause of larvae death (Koch’s postulates). Thereafter, the 7 outperforming isolates in their mortality rates against the insect were selected for molecular identification and a second round of bioassay (Figure 2). Out of the 39 tested isolates, 6 isolates (≈15%) achieved 100% mortality rate by the end of the experiment, 3 isolates (≈8%) scored 80% mortality, 6 isolates (≈15%) scored 60% mortality rate in *S. litura* larvae, while the remaining 24 isolates (≈61%) scored lower mortality scores (40–0%).

Therefore, based on the obtained results of this first bioassay, isolates TA-2, TA-4, KZ-2, KSH-2, KSH-8, DM-3 and DM-6 were selected for molecular identification and a second bioassay.

### 3.2. Molecular Identification and Phylogenetic Analysis

**Figure 3:**
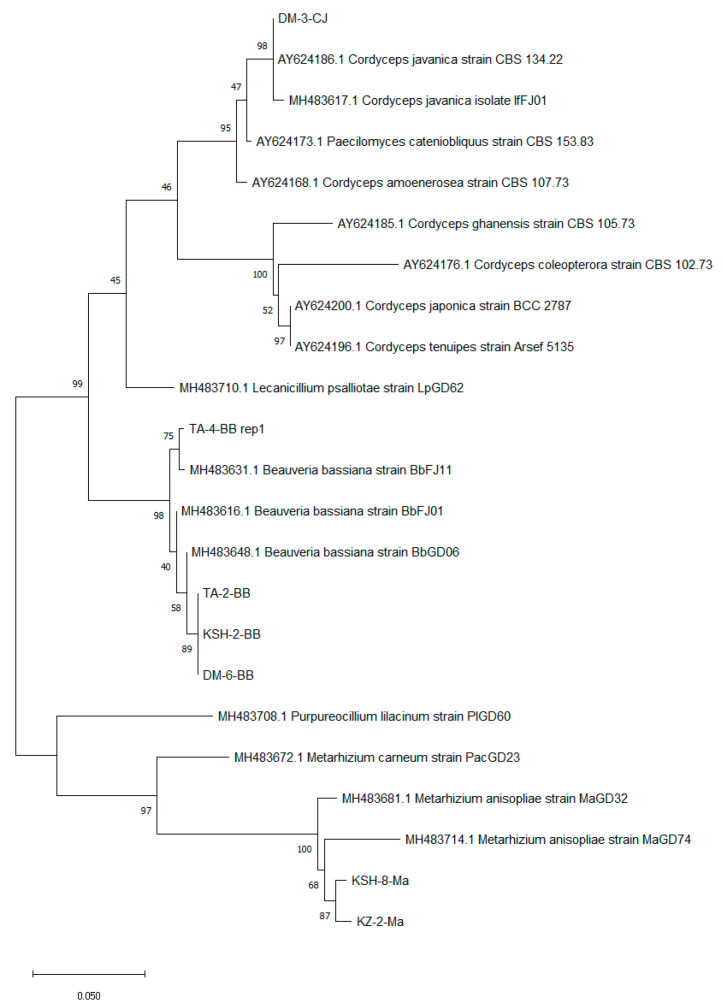
Molecular phylogeny analysis of selected entomopathogenic fungi based on molecular characteristics of ITS region in fungal DNA. The tree was inferred using the maximum likelihood method and Jukes–Cantor model in MEGA11. The percentage of trees in which the associated taxa clustered together is shown next to the branches. Initial trees for the heuristic search were obtained automatically by applying the neighbor-joining and BioNJ algorithms to a matrix of pairwise distances estimated using the Jukes–Cantor model, and then selecting the topology with superior log likelihood value. The tree is drawn to scale, with branch lengths measured in the number of substitutions per site. All positions containing gaps and missing data were eliminated and there was a total of 420 positions in the final dataset.

The ITS region was sequenced to identify selected fungal isolates and the results as shown in Figure 3, indicated that isolates TA-2, TA-4, KSH-2 and DM-6 were clustered with reference *B. bassiana* strains, and despite being isolated from different regions, isolates TA-2, KSH-2 and DM-6 were more closely related than TA-4 and other reference strains. Strains KSH-8 and KZ-2 were identified and clustered in the same clade with *Metarhizium anisopliae* and strain DM-3 was identified and clustered with *Cordyceps javanica* reference strains.

### 3.3. Second Round of Pathogenicity against S. litura and T. molitor

**Figure 4:**
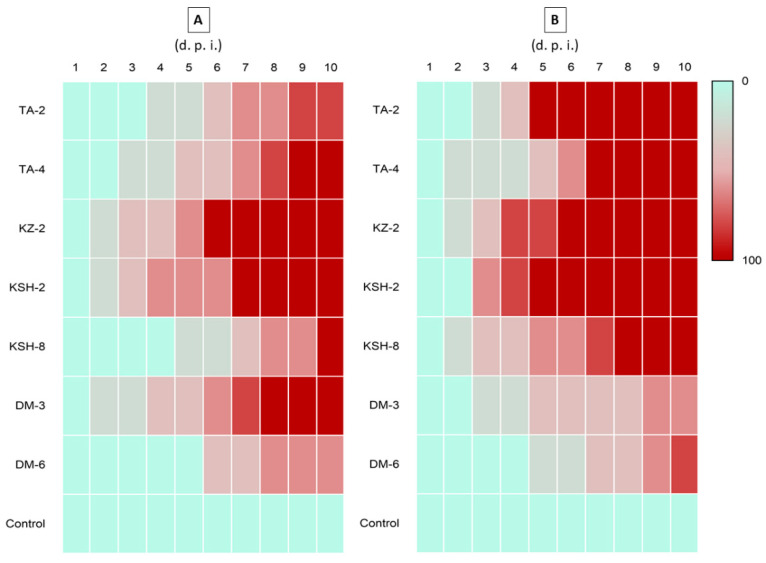
Insect killing heatmap of seven selected entomopathogenic fungal isolates belonging to three genera isolated from different regions in Nile Delta against *S. litura* (**A**) and *T. molitor* (**B**).

Selected isolates from the first pathogenicity bioassay belonging to three fungal genera displaying promising insecticidal activity under our test condition were further evaluated in a second pathogenicity screening against *S. litura* and *T. molitor.* The results presented in Figure 4 were in agreement with the first bioassay as five isolates (TA-4, KZ-2, KSH-2, KSH-8 and DM-3) scored 100% mortality, one isolate (TA-2) scored 80% and one isolate (DM-6) scored 60% killing rate of *S. litura* larvae under our test conditions. Meanwhile, the bioassay against *T. molitor* showed a similar outcome as the one against *S. litura* but with differences in isolate killing speed. Results showed that five isolates (TA-2, TA-4, KZ-2, KSH-2 and KSH-8) scored 100% mortality rate, one isolate (DM-6) scored 80% and one isolate (DM-3) scored 60% killing activity against *T. molitor*.

### 3.4. Thermotolerance Assay

**Figure 5:**
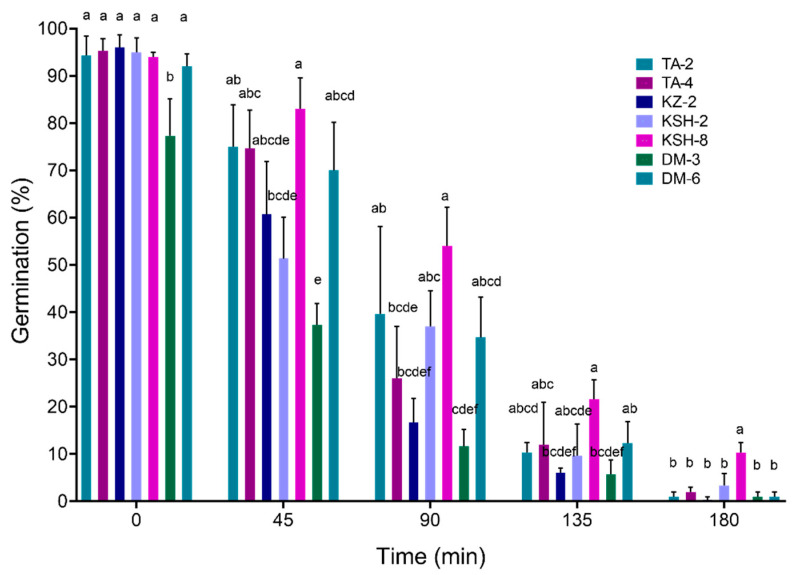
Conidial germination rate of selected fungal isolates after heat-treatments at 45 °C. Bars represent the average and standard deviation of 3 replicates (*n* = 3). Different letters (a–f) indicate significant differences between isolates for each timepoint (one-way ANOVA with Tukey HSD, *p* < 0.05).

For evaluating the effect of heat treatment on fungal conidial germination in selected isolates, conidial suspensions were exposed to 45 °C for different exposure periods (0, 45, 90, 135 and 180 min). The results presented in Figure 5 indicated that at the time point 0 min (before heat treatment), six isolates showed a high germination rate (˃92%) while only isolate, (DM-3), showed a significantly lower rate (≈77%) compared to the rest of the isolates. After exposure to 45 °C for different timepoints, germination rates decreased gradually with increasing exposure time. After the first time point (45 min), four isolates (TA-2, TA-4, KSH-8 and DM-6) displayed significantly higher tolerance (˃70% germination rate). At the 90 min time point, four isolates (TA-2, KSH-2, KSH-8 and DM-6) exhibited significantly higher tolerance. Moreover, at the time point of 135 min, the two isolates KZ-2 and DM-3 were significantly lower compared to the other isolates. Finally, only isolate KSH-8 displayed significantly higher tolerance after the 180 min treatment.

## 4. Discussion

Expansion and excessive use of chemical pesticides to control serious crop-attacking pathogens and insects have led to many negative side effects on human health and environmental biodiversity, including soil microorganisms [36]. Therefore, using beneficial microorganisms as biocontrol agents (BCAs) as an effective and ecofriendly alternative to chemical applications has emerged as a powerful tool with promising success under various experimental and field conditions [37,38,39]. This study aimed to isolate entomopathogenic fungal isolates indigenous to Nile Delta as potential BCAs to control important insects attacking crops and orchards in this vital area for Egypt’s food security. The insect baiting technique has been reported as a simple and effective tool for the isolation of insect associated EMPF from the soil [29,30] and DNA-based molecular characterization has become a standard approach for the accurate identification of microorganisms in this field [40]. Together they represent a pipeline for constructing collections of fungal isolates as essential step to develop microbial-based bioinsecticides as reported in numerous articles [13,41,42].

In this study, we demonstrated the efficacy of using insect baiting coupled with molecular identification and pathogenicity screening to isolate indigenous EMPF to control insect pests from Nile Delta soils (Figure S1). In this study we focused on the seven outperforming isolates under our test conditions. However, the potential value of other obtained isolates cannot be completely ignored and might require further evaluation under different experimental conditions in future work. Molecular identification and phylogenetic analysis revealed that selected isolates were identified as *B. bassiana*, *M. anisopliae* and *C. javanica*. *Beauveria* spp. are considered the most common cosmopolitan EMPF, parasitizing a wide range of insect species [10,13,43]. Both *B. bassiana* and *M. anisopliae* are among the most famous and globally distributed EMPF, revealing significant genetic diversification [44,45]. The versatile lifestyle of those two important EMPFs, wide host range and persistence in harsh habitats where preferred hosts (insects or plants) are temporarily unavailable, supported potential roles and values as microbial-based applications in integrated pest management strategies [45,46]. In agreement with our results, *B. bassiana* and *M. anisopliae* were isolated from soil samples from Assiut governorate (middle area of Egypt) using the *G. mellonella* baiting method [22]. Similarly, EMPF were obtained from soils of various locations in Egypt and virulence assays confirmed the efficacy of four *B. bassiana* and *M. anisopliae* isolates against red spider mites *Tetranychus urticae* (Koch) (Acari: Tetranychidae) [24]. Moreover, the insect baiting method using larvae of the Mediterranean flour moth, *Anagasta kuehniella* (Zeller) (Lepidoptera: Pyralidae) was used to entrap EMPF in agriculture soils from the Suez Canal area in the northeastern part of Egypt [47]. Isolated pathogens *B. bassiana, M. anisopliae, Paecilomyces fumosoroseus* and *Lecanicillium lecanii* were reported as the most virulent bioagents.

In this study, isolate (DM-3) was identified as *C. javanica,* which is a well-known insect-pathogen that has been extensively used to control a wide range of insect hosts [48,49,50]. In support of our results, *C. javanica* isolated from Korean soils using *G. mellonella* entrapment was reported to have dual biocontrol potential against the green peach aphid, *Myzus persicae* (Sulzer) (Hemiptera: Aphididae) and against both the fungal and oomycete pathogens *Colletotrichum gloeosporioides* and *Phytophthora capsici,* respectively [51]. Similarly, *C. javanica* was isolated from soil samples in China using a selective medium and was reported to have high efficacy against *Bemisia tabaci* (Gennadius) and *S. litura* [52]. To the best of our knowledge, this is the first report of *C. javanica* from Egyptian soils. In the second bioassay against *T. molitor*, selected isolates displayed high virulence suggesting a wide spectrum of insecticidal activity against coleopteran, lepidopteran and possibly other orders of insects. Finally, given that microbial-based formulations of BCAs are exposed to ever-changing conditions in open fields that may interfere and reduce their efficacy, it is of great importance to profile environmental persistence of selected EMPF against undermining factors such as elevated temperature, changing soil pH and/or exposure to UV irradiation, among many other influencing factors. The temperature of Nile Delta regions ranges between 32–38 °C, rarely reaching 45 °C in summer. Meanwhile, in winter the average temperature is 9 °C at night and increases to an average 19 °C during the daytime [53]. Therefore, in this study the thermotolerance activity of conidial suspensions was evaluated as a parameter for environmental stress tolerance. The results of thermotolerance assay revealed that increasing the heat treatment drastically decreased conidial germination rates. Nevertheless, some isolates were more tolerant than others at different time points of the heat treatment. The low thermotolerance potential of isolate DM-3 (*C. javanica*) compared to the other isolates might explain the scarcity of reports on this fungus from Egyptian soils. On the other hand, and in agreement with our results, numerous articles reported high thermotolerance of *B. bassiana* and *M. anisopliae* [41,54,55]. The variation in abiotic stress tolerance between EMPF isolates in this study as well as other studies indicates the importance of such factors, along with virulence, when considering EMPF for biocontrol under different environmental conditions.

In the future, further evaluation of other important growth characteristics might provide more information on potential efficacy of those and other EMPF isolates under field conditions.

## Data Availability

Not applicable.

## Supplementary Materials

The following are available online at https://www.mdpi.com/article/10.3390/jof8010054/s1, Figure S1: Graphical abstract of this study, Table S1: NCBI GenBank accessions for reference entomopathogenic fungi used in this study, Table S2: NCBI GenBank accessions for entomopathogenic fungi obtained during this study.
