# Supplementary material for: Screening and Identification of Indigenous Entomopathogenic Fungal Isolates from Agricultural Farmland Soils in Nile Delta, Egypt
Source: J Fungi (Basel). 2022 Jan 5;8(1):54. doi: 10.3390/jof8010054 (PMC8778751; doi:10.3390/jof8010054)
Supplement: Supplementary file 1 [file jof-08-00054-s001.zip › jof-1515758-supplementary.pdf]

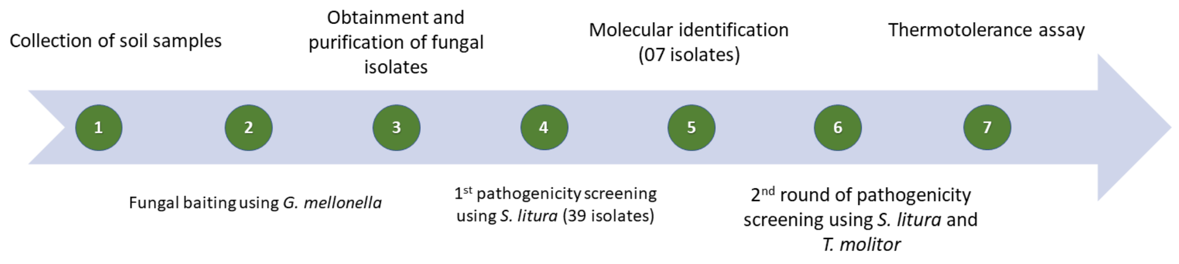


**Figure S1.** Graphical abstract of this study

**Table S1.** NCBI GenBank accessions for entomopathogenic fungi used in this study

| Fungal identification | Strain | Accession number | Reference |
|---|---|---|---|
| *Beauveria bassiana* | BbGD06 | MH483648.1 | (Niu et al., 2019) |
| *Cordyceps amoenerosea* | CBS 107.73 | AY624168.1 | (Luangsa-Ard et al., 2005) |
| *Purpureocillium lilacinum* | PlGD60 | MH483708.1 | (Niu et al., 2019) |
| *Metarhizium anisopliae* | MaGD32 | MH483681.1 | (Niu et al., 2019) |
| *Metarhizium carneum* | PacGD23 | MH483672.1 | (Niu et al., 2019) |
| *Beauveria bassiana* | BbGD06 | MH483648.1 | (Niu et al., 2019) |
| *Beauveria bassiana* | BbFJ11 | MH483631.1 | (Niu et al., 2019) |
| *Lecanicillium psalliotae* | LpGD62 | MH483710.1 | (Niu et al., 2019) |
| *Cordyceps javanica* | IfFJ01 | MH483617.1 | (Niu et al., 2019) |
| *Beauveria bassiana* | BbFJ01 | MH483616.1 | (Niu et al., 2019) |
| *Metarhizium anisopliae* | MaGD74 | MH483714.1 | (Niu et al., 2019) |
| *Cordyceps japonica* | BCC 2787 | AY624200.1 | (Luangsa-Ard et al., 2005) |
| *Cordyceps tenuipes* | Arsef 5135 | AY624196.1 | (Luangsa-Ard et al., 2005) |
| *Cordyceps javanica* | CBS 134.22 | AY624186.1 | (Luangsa-Ard et al., 2005) |
| *Cordyceps ghanensis* | CBS 105.73 | AY624185.1 | (Luangsa-Ard et al., 2005) |
| *Cordyceps coleopterora* | CBS 102.73 | AY624176.1 | (Luangsa-Ard et al., 2005) |
| *Paecilomyces catenioblíquus* | CBS 153.83 | Y624173.1 | (Luangsa-Ard et al., 2005) |

**Table S2.** NCBI GenBank accessions for selected entomopathogenic fungi obtained during this study

| Fungal identification | Strain | Site | Accession number |
|---|---|---|---|
| *Beauveria bassiana* | TA-2 | Tanta | OL631250 |
| *Metarhizium anisopliae* | KZ-2 | Kafr El-Zayat | OL631251 |
| *Metarhizium anisopliae* | KSH-8 | Kafr El-Shaikh | OL631252 |
| *Beauveria bassiana* | KSH-2 | Kafr El-Shaikh | OL631253 |
| *Beauveria bassiana* | TA-4 | Tanta | OL631254 |
| *Beauveria bassiana* | DM-6 | Damietta | OL631255 |
| *Cordyceps javanica* | DM-3 | Damietta | OL631256 |
